# Supplementary material for: Delayed induction of type I and III interferons mediates nasal epithelial cell permissiveness to SARS-CoV-2
Source: Nat Commun. 2021 Dec 7;12:7092. doi: 10.1038/s41467-021-27318-0 (PMC8651658; doi:10.1038/s41467-021-27318-0)
Supplement: Supplementary file 3 — Description of Additional Supplementary Files [file 41467_2021_27318_MOESM3_ESM.pdf]

Description of Additional Supplementary Files

File Name: Supplementary Data 1
Description: Data for characterisation of nasal cells by clustering. Single-cell RNA-seq libraries were generated from two representative donors, yielding 28,346 individual transcriptomes for analysis following quality control. Following dimensionality reduction and Leiden clustering, eight populations were discerned by their expression of established marker genes. Cells were examined for expression of characteristic markers.

File Name: Supplementary Data 2
Description: Differential expression (DE) analysis between infected cluster. Wilcoxon rank sum test, two-sided with multiple comparisons, was undertaken to compare mock-infected cell transcriptomes with those from bystander cells (without detectable viral transcripts) and infected cells (with detectable viral transcripts) from the virus-exposed cultures (n=2 donors per condition, MOI 0.1, 24 hours post infection).

File Name: Supplementary Data 3
Description: Statistical data from the differential gene expression analysis between the untreated and bystander cells in the different cell types (Wilcoxon rank sum test, one vs rest, $p < 0.05$, n=2 donors per condition, MOI 0.1, 24 hours post infection).

File Name: Supplementary Data 4
Description: Statistical data from the differential gene expression analysis between the untreated and infected cells in the different cell types (Wilcoxon rank sum test, one vs rest, $P < 0.05$, n=2 donors per condition, MOI 0.1, 24 hours post infection).

File Name: Supplementary Data 5
Description: Data from the differential proteomic profiling of SARS-CoV-2-infected nasal ALI cultures. Mass spectrometry-based proteomics was carried out on whole-cell lysates prepared at 72 hours post infection (n=6 donors per condition, MOI 0.1). Proteins that displayed a significant increase or decrease are listed with the exact adjusted p values and logFC values. This includes the metadata, exact P values, heat map data and MQ output.

File Name: Supplementary Data 6
Description: Summary data from composite interferon-stimulated gene (ISG) signature score violin plot values. This includes median, standard deviation and interquartile values. and the exact P values from the analysis (Wilcoxon rank sum test, one vs rest, n=2 donors per condition, MOI 0.1, 24 hours post infection).
