# Supplementary material for: Delayed induction of type I and III interferons mediates nasal epithelial cell permissiveness to SARS-CoV-2
Source: Nat Commun. 2021 Dec 7;12:7092. doi: 10.1038/s41467-021-27318-0 (PMC8651658; doi:10.1038/s41467-021-27318-0)
Supplement: Supplementary file 10 — Reporting Summary [file 41467_2021_27318_MOESM10_ESM.pdf]

nature research

Corresponding author(s): Dr. Christopher JA Duncan

Last updated by author(s): 2021/10/10

Reporting Summary

Nature Research wishes to improve the reproducibility of the work that we publish. This form provides structure for consistency and transparency in reporting. For further information on Nature Research policies, see our Editorial Policies and the Editorial Policy Checklist.

Statistics

For all statistical analyses, confirm that the following items are present in the figure legend, table legend, main text, or Methods section.

n/a	Confirmed	
☐	☒	The exact sample size (*n*) for each experimental group/condition, given as a discrete number and unit of measurement
☐	☒	A statement on whether measurements were taken from distinct samples or whether the same sample was measured repeatedly
☐	☒	The statistical test(s) used AND whether they are one- or two-sided<br>*Only common tests should be described solely by name; describe more complex techniques in the Methods section.*
☒	☐	A description of all covariates tested
☐	☒	A description of any assumptions or corrections, such as tests of normality and adjustment for multiple comparisons
☐	☒	A full description of the statistical parameters including central tendency (e.g. means) or other basic estimates (e.g. regression coefficient) AND variation (e.g. standard deviation) or associated estimates of uncertainty (e.g. confidence intervals)
☐	☒	For null hypothesis testing, the test statistic (e.g. F, t, r) with confidence intervals, effect sizes, degrees of freedom and P value noted<br>*Give P values as exact values whenever suitable.*
☒	☐	For Bayesian analysis, information on the choice of priors and Markov chain Monte Carlo settings
☒	☐	For hierarchical and complex designs, identification of the appropriate level for tests and full reporting of outcomes
☐	☒	Estimates of effect sizes (e.g. Cohen's d, Pearson's r), indicating how they were calculated

Our web collection on statistics for biologists contains articles on many of the points above.

Software and code

Policy information about availability of computer code

Data collection

Software:
The following software packages were used for data collection:
Image Studio v5.2.5 (LI-COR): immunoblotting
Radius software v2.1 (EMSIS): electron micrographs
NIS-Elements C Software: immunofluorescence micrographs
Agilent Aria Mx software v1.6: qRT-PCR
Novaseq Control Software v1.7: RNA sequencing
UltiMate 3000 RSLCnano systems software: LCMS
Radius software v2.1: digital micrographs
Detailed parameters of each of the methods are mentioned in relevant sections in Methods.

Code:
Analysis scripts and codes are available at github.com/haniffalab/covid_nasal_epithelium. No custom code was created for the purpose of data collection.

Data analysis

Software:
The following software packages were used for data analysis:
Image Studio v5.2.5 (LI-COR): immunoblotting
ImageJ v2.1.0 (Fiji): immunofluorescence (Cell Counter and Plot Profile plugins) and TEM analysis
MaxQuant v1.6.10.43: proteomics
STRING database v11: proteomics differential abundance
Cytoscape v3.7.2: proteomics presentation

CellRanger v4.0: scRNA-seq data alignment and analysis
CellBender v0.2.0: scRNA-seq data
GraphPad prism v9.0: statistical analysis and figure preparation
R studio v3.6.2: proteomics (Limma) and scRNA-seq data analysis and figure preparation. Additional packages in R studio were used including: Scrublet v0.2.1, Seurat v4.0.1 - including label transfer, AddModuleScore tools, FindAllMarkers function, Harmony v1, fgsea v1.10.1, Leiden algorithm v0.3.9, DoRoTHea v1.4.2, VIPER v1.24.0.
Detailed parameters of each of the methods are mentioned in relevant sections in Methods.

Code:
Analysis scripts and codes are available at github.com/haniffalab/covid_nasal_epithelium. No custom code was created for the purpose of data analysis.

For manuscripts utilizing custom algorithms or software that are central to the research but not yet described in published literature, software must be made available to editors and reviewers. We strongly encourage code deposition in a community repository (e.g. GitHub). See the Nature Research guidelines for submitting code & software for further information.

Data

Policy information about availability of data

All manuscripts must include a data availability statement. This statement should provide the following information, where applicable:
- Accession codes, unique identifiers, or web links for publicly available datasets
- A list of figures that have associated raw data
- A description of any restrictions on data availability

Source data are provided with this paper. List of figures with associated raw data: Fig. 1a-h, Fig. 2d-e, Fig. 3a-c Fig. 4a-f, Fig. 5a-c, Fig. 6a-e, Fig. 7a-e, and supplementary Fig. 2, supplementary Fig. 7, supplementary Fig. 8, supplementary Fig. 10. Source data includes uncropped blots, all quantitative data and PFU counts. The results of differential expression analysis of RNA-seq and proteomics data are included as supplementary datasets. Additional raw data are available on request from the corresponding author providing ethical approvals permit sharing of data.
The mass spectrometry proteomics data generated in this study have been deposited to the ProteomeXchange Consortium via the PRIDE partner repository with the dataset identifier PXD022523. This can be accessed through the Username: reviewer_pxd022523@ebi.ac.uk and the Password: UaEXYFKF. The proteomic analysis in this study involved using reference proteomes from the following databases: SwissProt Homo sapiens under the proteome ID: UP000005640 9606 and Trembl SARS-CoV-2 FASTA files under the proteome ID: UP000464024.
Raw RNA sequencing data have been deposited to the European Genome-Phenome Archive (study ID:) EGAS00001005633. Processed scRNAseq data is available at Zenodo (https://zenodo.org/record/4564332, DOI: 10.5281/zenodo.4564332). RNA Sequencing data was aligned to the combined human transcriptome using the official Cell Ranger reference under the assession code GRCh38-2020-A (https://support.10xgenomics.com/single-cell-gene-expression/software/release-notes/build#GRCh38_2020A) and the SARS-CoV-2 reference transcriptomes are available in Ensembl under the reference code Sars_cov_2.ASM985889v3 (https://covid-19.ensembl.org/index.html). The clustering annotation was validated using Seurat label transfer from a published scRNA-seq dataset from nasopharyngeal swabs (DOI: 10.1016/j.cell.2021.07.023). ISG signature scores were generated using context-specific ISGs from a published IFN-treated nasal cell dataset (DOI: 10.1016/j.cell.2020.04.035).

Field-specific reporting

Please select the one below that is the best fit for your research. If you are not sure, read the appropriate sections before making your selection.

[x] Life sciences [ ] Behavioural & social sciences [ ] Ecological, evolutionary & environmental sciences

For a reference copy of the document with all sections, see nature.com/documents/nr-reporting-summary-flat.pdf

Life sciences study design

All studies must disclose on these points even when the disclosure is negative.

Sample size	Human nasal epithelial cells from 6 independent donors were used in this study. Sample sizes selected were typical for studies of this nature, based on our prior experience of studies in this model, and ensured adequate statistical power to ensure detect relevant differences.
Data exclusions	No data were excluded from the analysis.
Replication	All experimental data were reproducible and unless otherwise stated all experiments were successfully repeated in more than one independent donor.
Randomization	No randomisation steps were performed for experiments or data analysis. Randomisation is not relevant to the design of this in vitro basic science study.
Blinding	Investigators were blinded to SARS-CoV-2 infection status for TEM image collection. Otherwise no blinding was used as it was not relevant since there was no allocation to treatment groups.

Reporting for specific materials, systems and methods

We require information from authors about some types of materials, experimental systems and methods used in many studies. Here, indicate whether each material, system or method listed is relevant to your study. If you are not sure if a list item applies to your research, read the appropriate section before selecting a response.

Materials & experimental systems

n/a	Involved in the study
☐	☒ Antibodies
☐	☒ Eukaryotic cell lines
☒	☐ Palaeontology and archaeology
☒	☐ Animals and other organisms
☐	☒ Human research participants
☒	☐ Clinical data
☒	☐ Dual use research of concern

Methods

n/a	Involved in the study
☒	☐ ChIP-seq
☒	☐ Flow cytometry
☒	☐ MRI-based neuroimaging

Antibodies

Antibodies used

Antibody manufacturer and catalogue numbers. Clone and lot number are provided when possible. (catalogue numbers = cat#, lot number = lot#)
Primary antibodies: Spike (Novus, cat# nb100-56578, lot# AB092903C-08), RSAD2 (CST, cat# 13996, clone = D5T2X, lot = 1, 1:1000 dilution), ISG15 (CST, cat# 2743, lot=3, 1:1000 dilution), USP18 (SCB, cat# sc-1668, clone# D4E7, lot# 1, 1:2000 dilution), ACE2 (Abcam, cat# ab15348, lot# GR3333640-4, 1:1000 dilution), ACE2 (R&D, cat# AF933, lot# HOK0320032, 1:200 dilution), TMPRSS2 (Abcam, cat# ab92323, lot# GR3344246-1, 1:1000 dilution), MxA (SCB, cat# sc-50509, clone# H-285, 1:1000 dilution), GAPDH (CST, cat# 5174, clone# D16H11, lot# 8, 1:10000 dilution), MUC5B (Sigma, cat# HPA008246, 1:1000 dilution), MUC5AC (Sigma, cat# HPA040615), TP63 (Abcam, cat# ab735, clone# 4A4), Acetylated-alpha tubulin (Abcam, ab24610, 6-11B-1)
Secondary antibodies:, Anti-rabbit HRP- conjugated (CST, cat# 7074, lot# 29), Anti-mouse HRP-conjugated (CST, cat# 7076, lot# 33),, AF488 conjugated anti-mouse (TFS, cat# A-11001 , lot# 2090562), AF488 conjugated anti-rabbit Goat (TFS, cat# A-11008, clone# G-A-5), AF594 conjugated anti-mouse (TFS, cat# A-11005, lot# 2234977), AF594 conjugated anti-rabbit Goat (TFS, cat# A-11012, lot# 2266563).

Validation

All antibodies are commercially available and have been validated by the manufacturer, as stated on the manufacturers website, and internally validated for use in this project. Viral antibody (Spike) specificity was validated by comparing mock versus infected vero E6 cells. Cell marker antibodies (e.g. Ace2, TMPRSS2, MUC5AC, MUC5B, TP63) were validated by comparing the nasal ALIs to non-expressing cells (e.g. macrophages). Immune makers (e.g. RSAD2, USP18, ISG15, MxA) were validated using fibroblasts treated with a stimulant (i.e. IFNa) compared to untreated cells.

Eukaryotic cell lines

Policy information about cell lines

Cell line source(s)	Vero E6 cells (ATCC CRL-1586) and MDCK cells (ATCC CCL-34) were obtained from the American tissue culture consortium.
Authentication	The cell lines were not authenticated
Mycoplasma contamination	Cell lines were not tested for mycoplasma contamination.
Commonly misidentified lines (See ICLAC register)	No commonly misidentified lines were used

Human research participants

Policy information about studies involving human research participants

Population characteristics

The population consisted of equal numbers of males and females, between the ages of 38 and 78 (see supplementary Table S3 for more details). Additional information is not available on other demographics beyond age and sex and is not relevant to the analyses performed.

Recruitment

Adult primary human nasal airway epithelial cells were derived from excess clinical material obtained from patients recruited during routine nasal surgical procedures. Written informed consent was obtained prior to sample collection. Access to tissue for this study was provided by the Newcastle Biobank which is supported by the Newcastle upon Tyne NHS Foundation Trust and Newcastle University. No significant source of bias is identified. Participants were not compensated for their sample donation.

Ethics oversight

Ethical approval for sample collection was provided via the Newcastle and North Tyne Research Ethics Committee (Reference 17/NE/0361) and written informed consent was provided prior to sample collection.

Note that full information on the approval of the study protocol must also be provided in the manuscript.
